# 7-Chloro-4-phenethyl-2*H*-1,4-benzoxazin-3(4*H*)-one

**DOI:** 10.1107/S1600536809007739

**Published:** 2009-03-06

**Authors:** Ming-Jun Chen, Hao Yang, Wen-Liang Dong, Hua Zuo, Jia-Zhou Zhou

**Affiliations:** a College of Chemical and Environmental Engineering, Chongqing Three Gorges University, Chongqing 404000, People’s Republic of China; b College of Horticulture and Landscape Architecture, Southwest University, Chongqing 400715, People’s Republic of China; c School of Pharmaceutical Sciences, Shandong University of Traditional Chinese Medicine, Jinan 250355, People’s Republic of China; d College of Pharmaceutical Sciences, Southwest University, Chongqing 400716, People’s Republic of China

## Abstract

In the crystal structure of title compound, C_16_H_14_ClNO_2_, the dihedral angle between the aromatic rings is 4.2 (2)°.

## Related literature

For related structures, see: Li *et al.* (2008 ▶); Zuo *et al.* (2008 ▶).
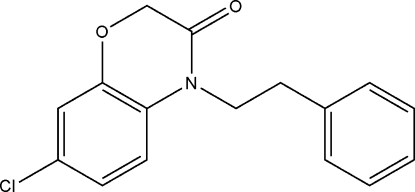


## Experimental

### 

#### Crystal data

C_16_H_14_ClNO_2_
*M* *_r_* = 287.73
Orthorhombic, 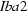

*a* = 13.528 (4) Å
*b* = 29.616 (10) Å
*c* = 7.074 (2) Å
*V* = 2834.2 (15) Å^3^
*Z* = 8
Mo *K*α radiation
μ = 0.27 mm^−1^
*T* = 298 K
0.12 × 0.10 × 0.06 mm

#### Data collection

Bruker SMART CCD area-detector diffractometer
Absorption correction: multi-scan (*SADABS*; Bruker, 2005 ▶) *T*_min_ = 0.968, *T*_max_ = 0.984
7036 measured reflections
2171 independent reflections
1343 reflections with *I* > 2σ(*I*)
*R*_int_ = 0.067

#### Refinement

*R*[*F* ^2^ > 2σ(*F* ^2^)] = 0.049
*wR*(*F* ^2^) = 0.117
*S* = 1.00
2171 reflections
182 parameters
H-atom parameters constrained
Δρ_max_ = 0.15 e Å^−3^
Δρ_min_ = −0.16 e Å^−3^
Absolute structure: Flack (1983 ▶), 797 Friedel pairs
Flack parameter: 0.04 (13)

### 

Data collection: *SMART* (Bruker, 2005 ▶); cell refinement: *SAINT* (Bruker, 2005 ▶); data reduction: *SAINT*; program(s) used to solve structure: *SHELXS97* (Sheldrick, 2008 ▶); program(s) used to refine structure: *SHELXL97* (Sheldrick, 2008 ▶); molecular graphics: *XP* in *SHELXTL* (Sheldrick, 2008 ▶); software used to prepare material for publication: *SHELXL97*.

## Supplementary Material

Crystal structure: contains datablocks I, global. DOI: 10.1107/S1600536809007739/nc2136sup1.cif

Structure factors: contains datablocks I. DOI: 10.1107/S1600536809007739/nc2136Isup2.hkl

Additional supplementary materials:  crystallographic information; 3D view; checkCIF report

## Figures and Tables

**Fig. 1.**
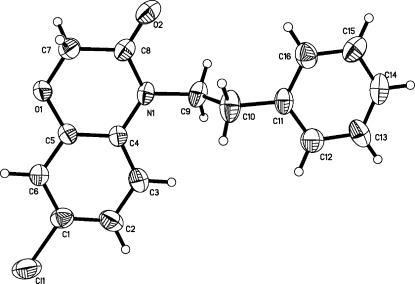
The molecular structure of the title compound with labelling and displacement ellipsoids drawn at the 50% probability level.

## supplementary crystallographic information

### Comment

As part of our continuing project on the study of the interactions occurring between small molecules and proteins (Li *et al.*, 2008; Zuo *et al.*, 2008), we report here the synthesis and crystal structure of the title compound. In the crystal structure, the two ring systems are nearly coplanar, the diehderal angle between the aromatic rings being 4.2 (2)°.

### Experimental

To the solution of 2-(2,4-dichlorophenoxy)-*N*-phenethylacetamide (0.684 g, 2.0 mmol) in DMF (20 ml), caesium carbonate (0.787 g, 2.4 mmol) was added. The mixture was refluxed for 1.5 h. After completion of the reaction (by TLC monitoring), the DMF was removed under vacuum. Water (20 ml) was added into the residue to obtain a turbid solution and it was extracted by ethyl acetate (20 ml *x* 4). The combined organic layers were washed three times with 10 mL of 1 mol/*L* hydrochloric acid and saturated sodium chloride solution (10 ml *x* 3), dried over MgSO_4_. And then the mixture was filtered and the filtrate obtained was concentrated under reduced pressure to obtain the corresponding crude product. The product was purified by column chromatography on silica gel using ethyl/acetate = 1/5 as eluent (yield 75%). Crystals suitable for X-ray diffraction were obtained by slow evaporation of a solutionof the solid dissolved in ethyl acetate/hexane at room temperature for 10 days.

### Refinement

All H atoms were palced in calculated positions and refined as riding, with C—H = 0.93–0.97\%A, and with *U*_iso_(H)=1.2Ueq(C). The absolute structure was determined on the basis of 797Friedel pairs.

### Crystal data

**Table:**

C_16_H_14_ClNO_2_	*F*(000) = 1200
*M**_r_* = 287.73	*D*_x_ = 1.349 Mg m^−^^3^
Orthorhombic, *I**b**a*2	Mo *K*α radiation, λ = 0.71073 Å
Hall symbol: I 2 -2c	Cell parameters from 809 reflections
*a* = 13.528 (4) Å	θ = 2.6–18.3°
*b* = 29.616 (10) Å	µ = 0.27 mm^−^^1^
*c* = 7.074 (2) Å	*T* = 298 K
*V* = 2834.2 (15) Å^3^	Block, colorless
*Z* = 8	0.12 × 0.10 × 0.06 mm

### Data collection

**Table:**

Bruker SMART CCD area-detector diffractometer	2171 independent reflections
Radiation source: fine-focus sealed tube	1343 reflections with *I* > 2σ(*I*)
graphite	*R*_int_ = 0.067
φ and ω scans	θ_max_ = 25.0°, θ_min_ = 1.7°
Absorption correction: multi-scan (*SADABS*; Bruker, 2005)	*h* = −13→16
*T*_min_ = 0.968, *T*_max_ = 0.984	*k* = −34→34
7036 measured reflections	*l* = −8→7

### Refinement

**Table:**

Refinement on *F*^2^	Hydrogen site location: inferred from neighbouring sites
Least-squares matrix: full	H-atom parameters constrained
*R*[*F*^2^ > 2σ(*F*^2^)] = 0.049	*w* = 1/[σ^2^(*F*_o_^2^) + (0.04*P*)^2^ + 1.1309*P*] where *P* = (*F*_o_^2^ + 2*F*_c_^2^)/3
*wR*(*F*^2^) = 0.117	(Δ/σ)_max_ < 0.001
*S* = 1.00	Δρ_max_ = 0.15 e Å^−^^3^
2171 reflections	Δρ_min_ = −0.16 e Å^−^^3^
182 parameters	Extinction correction: *SHELXL97* (Sheldrick, 2008), Fc^*^=kFc[1+0.001xFc^2^λ^3^/sin(2θ)]^-1/4^
0 restraints	Extinction coefficient: 0.0057 (5)
Primary atom site location: structure-invariant direct methods	Absolute structure: Flack (1983), 797 Friedel pairs
Secondary atom site location: difference Fourier map	Flack parameter: 0.04 (13)

### Special details

**Table:**

Geometry. All e.s.d.'s (except the e.s.d. in the dihedral angle between two l.s. planes) are estimated using the full covariance matrix. The cell e.s.d.'s are taken into account individually in the estimation of e.s.d.'s in distances, angles and torsion angles; correlations between e.s.d.'s in cell parameters are only used when they are defined by crystal symmetry. An approximate (isotropic) treatment of cell e.s.d.'s is used for estimating e.s.d.'s involving l.s. planes.
Refinement. Refinement of *F*^2^ against ALL reflections. The weighted *R*-factor *wR* and goodness of fit *S* are based on *F*^2^, conventional *R*-factors *R* are based on *F*, with *F* set to zero for negative *F*^2^. The threshold expression of *F*^2^ > σ(*F*^2^) is used only for calculating *R*-factors(gt) *etc*. and is not relevant to the choice of reflections for refinement. *R*-factors based on *F*^2^ are statistically about twice as large as those based on *F*, and *R*- factors based on ALL data will be even larger.The absolute structure was determined on the basis of 800 Friedel pairs.

### Fractional atomic coordinates and isotropic or equivalent isotropic displacement parameters (Å^2^)

**Table:**

	*x*	*y*	*z*	*U*_iso_*/*U*_eq_	
Cl1	0.42978 (9)	0.23193 (3)	−0.4987 (3)	0.1035 (6)	
O1	0.4487 (2)	0.39965 (8)	−0.4216 (4)	0.0762 (10)	
O2	0.3501 (2)	0.47560 (9)	−0.0754 (4)	0.0776 (10)	
N1	0.3137 (2)	0.40207 (11)	−0.1198 (5)	0.0524 (9)	
C1	0.3939 (3)	0.28154 (13)	−0.3869 (7)	0.0641 (12)	
C2	0.3273 (4)	0.28028 (16)	−0.2421 (9)	0.0824 (15)	
H2	0.3002	0.2530	−0.2033	0.099*	
C3	0.3007 (3)	0.32009 (15)	−0.1536 (7)	0.0754 (13)	
H3	0.2548	0.3193	−0.0557	0.091*	
C4	0.3405 (3)	0.36100 (13)	−0.2074 (6)	0.0489 (10)	
C5	0.4080 (3)	0.36097 (13)	−0.3538 (6)	0.0520 (10)	
C6	0.4356 (3)	0.32152 (12)	−0.4442 (6)	0.0586 (11)	
H6	0.4815	0.3221	−0.5420	0.070*	
C7	0.4403 (3)	0.43887 (13)	−0.3107 (7)	0.0608 (11)	
H7A	0.4279	0.4640	−0.3956	0.073*	
H7B	0.5041	0.4442	−0.2523	0.073*	
C8	0.3644 (3)	0.44065 (14)	−0.1590 (6)	0.0556 (11)	
C9	0.2368 (3)	0.40386 (14)	0.0274 (6)	0.0640 (11)	
H9A	0.1854	0.3822	−0.0027	0.077*	
H9B	0.2071	0.4337	0.0278	0.077*	
C10	0.2775 (3)	0.39363 (18)	0.2246 (7)	0.0811 (14)	
H10A	0.3003	0.3626	0.2285	0.097*	
H10B	0.3338	0.4130	0.2491	0.097*	
C11	0.2016 (3)	0.40062 (16)	0.3760 (6)	0.0581 (11)	
C12	0.1471 (3)	0.36580 (15)	0.4534 (7)	0.0721 (12)	
H12	0.1573	0.3364	0.4120	0.087*	
C13	0.0770 (3)	0.37441 (18)	0.5926 (8)	0.0776 (14)	
H13	0.0415	0.3507	0.6453	0.093*	
C14	0.0605 (3)	0.4166 (2)	0.6510 (7)	0.0797 (14)	
H14	0.0122	0.4219	0.7419	0.096*	
C15	0.1123 (4)	0.45149 (17)	0.5808 (7)	0.0819 (14)	
H15	0.1010	0.4807	0.6240	0.098*	
C16	0.1827 (3)	0.44326 (15)	0.4432 (7)	0.0724 (12)	
H16	0.2185	0.4674	0.3946	0.087*	

### Atomic displacement parameters (Å^2^)

**Table:**

	*U*^11^	*U*^22^	*U*^33^	*U*^12^	*U*^13^	*U*^23^
Cl1	0.0963 (9)	0.0644 (7)	0.1497 (14)	−0.0060 (7)	0.0085 (10)	−0.0330 (9)
O1	0.110 (2)	0.0537 (15)	0.064 (2)	−0.0103 (15)	0.038 (2)	−0.0038 (16)
O2	0.088 (2)	0.0731 (19)	0.072 (3)	0.0146 (16)	0.0006 (19)	−0.0206 (18)
N1	0.048 (2)	0.071 (2)	0.038 (2)	0.0065 (17)	0.0033 (17)	−0.0021 (18)
C1	0.058 (2)	0.057 (2)	0.078 (3)	−0.002 (2)	−0.004 (3)	−0.009 (2)
C2	0.079 (3)	0.061 (3)	0.108 (4)	−0.015 (2)	0.015 (3)	0.005 (3)
C3	0.064 (3)	0.079 (3)	0.083 (3)	−0.010 (2)	0.022 (2)	0.005 (3)
C4	0.044 (2)	0.057 (2)	0.046 (2)	0.0034 (19)	0.000 (2)	0.005 (2)
C5	0.050 (2)	0.055 (2)	0.051 (3)	−0.0021 (19)	0.002 (2)	0.007 (2)
C6	0.054 (2)	0.060 (2)	0.062 (3)	0.000 (2)	0.009 (2)	−0.001 (2)
C7	0.064 (2)	0.058 (2)	0.061 (3)	0.006 (2)	0.003 (2)	−0.001 (2)
C8	0.054 (3)	0.064 (3)	0.049 (3)	0.011 (2)	−0.005 (2)	−0.006 (2)
C9	0.051 (2)	0.094 (3)	0.047 (3)	0.016 (2)	0.002 (2)	−0.004 (2)
C10	0.059 (2)	0.132 (4)	0.052 (3)	0.015 (3)	0.002 (2)	0.009 (3)
C11	0.048 (2)	0.087 (3)	0.039 (3)	0.006 (2)	−0.003 (2)	0.007 (2)
C12	0.083 (3)	0.074 (3)	0.059 (3)	0.000 (3)	−0.010 (3)	0.002 (3)
C13	0.071 (3)	0.097 (3)	0.065 (3)	−0.023 (3)	0.000 (3)	0.021 (3)
C14	0.063 (3)	0.127 (4)	0.049 (3)	0.001 (3)	0.006 (2)	0.000 (3)
C15	0.089 (3)	0.088 (3)	0.069 (3)	0.007 (3)	−0.001 (3)	−0.023 (3)
C16	0.070 (3)	0.082 (3)	0.066 (3)	−0.021 (2)	0.003 (3)	0.000 (3)

### Geometric parameters (Å, °)

**Table:**

Cl1—C1	1.737 (4)	C7—H7B	0.9700
O1—C5	1.359 (4)	C9—C10	1.529 (6)
O1—C7	1.406 (4)	C9—H9A	0.9700
O2—C8	1.208 (4)	C9—H9B	0.9700
N1—C8	1.361 (5)	C10—C11	1.498 (6)
N1—C4	1.413 (5)	C10—H10A	0.9700
N1—C9	1.473 (5)	C10—H10B	0.9700
C1—C2	1.365 (7)	C11—C16	1.373 (5)
C1—C6	1.372 (5)	C11—C12	1.381 (6)
C2—C3	1.383 (6)	C12—C13	1.390 (6)
C2—H2	0.9300	C12—H12	0.9300
C3—C4	1.380 (5)	C13—C14	1.335 (6)
C3—H3	0.9300	C13—H13	0.9300
C4—C5	1.381 (5)	C14—C15	1.344 (6)
C5—C6	1.383 (5)	C14—H14	0.9300
C6—H6	0.9300	C15—C16	1.383 (6)
C7—C8	1.486 (5)	C15—H15	0.9300
C7—H7A	0.9700	C16—H16	0.9300
			
C5—O1—C7	117.8 (3)	N1—C9—C10	112.6 (3)
C8—N1—C4	120.2 (3)	N1—C9—H9A	109.1
C8—N1—C9	118.0 (3)	C10—C9—H9A	109.1
C4—N1—C9	121.5 (3)	N1—C9—H9B	109.1
C2—C1—C6	121.1 (4)	C10—C9—H9B	109.1
C2—C1—Cl1	120.2 (4)	H9A—C9—H9B	107.8
C6—C1—Cl1	118.7 (4)	C11—C10—C9	112.2 (3)
C1—C2—C3	119.3 (4)	C11—C10—H10A	109.2
C1—C2—H2	120.4	C9—C10—H10A	109.2
C3—C2—H2	120.4	C11—C10—H10B	109.2
C4—C3—C2	121.5 (4)	C9—C10—H10B	109.2
C4—C3—H3	119.3	H10A—C10—H10B	107.9
C2—C3—H3	119.3	C16—C11—C12	116.7 (4)
C3—C4—C5	117.7 (4)	C16—C11—C10	120.1 (4)
C3—C4—N1	122.3 (4)	C12—C11—C10	123.1 (5)
C5—C4—N1	120.0 (3)	C11—C12—C13	120.5 (4)
O1—C5—C4	122.1 (4)	C11—C12—H12	119.7
O1—C5—C6	116.1 (4)	C13—C12—H12	119.7
C4—C5—C6	121.7 (4)	C14—C13—C12	120.4 (4)
C1—C6—C5	118.8 (4)	C14—C13—H13	119.8
C1—C6—H6	120.6	C12—C13—H13	119.8
C5—C6—H6	120.6	C13—C14—C15	121.2 (5)
O1—C7—C8	119.2 (3)	C13—C14—H14	119.4
O1—C7—H7A	107.5	C15—C14—H14	119.4
C8—C7—H7A	107.5	C14—C15—C16	118.9 (5)
O1—C7—H7B	107.5	C14—C15—H15	120.5
C8—C7—H7B	107.5	C16—C15—H15	120.5
H7A—C7—H7B	107.0	C11—C16—C15	122.3 (4)
O2—C8—N1	122.6 (4)	C11—C16—H16	118.9
O2—C8—C7	119.7 (4)	C15—C16—H16	118.9
N1—C8—C7	117.8 (4)		
			
C6—C1—C2—C3	0.8 (7)	C4—N1—C8—O2	−173.2 (4)
Cl1—C1—C2—C3	179.2 (4)	C9—N1—C8—O2	1.6 (6)
C1—C2—C3—C4	−0.6 (8)	C4—N1—C8—C7	6.6 (5)
C2—C3—C4—C5	0.3 (7)	C9—N1—C8—C7	−178.6 (3)
C2—C3—C4—N1	179.5 (4)	O1—C7—C8—O2	−172.8 (4)
C8—N1—C4—C3	171.0 (4)	O1—C7—C8—N1	7.4 (6)
C9—N1—C4—C3	−3.6 (6)	C8—N1—C9—C10	−89.3 (5)
C8—N1—C4—C5	−9.8 (5)	C4—N1—C9—C10	85.4 (5)
C9—N1—C4—C5	175.6 (3)	N1—C9—C10—C11	173.5 (4)
C7—O1—C5—C4	15.7 (5)	C9—C10—C11—C16	−81.2 (6)
C7—O1—C5—C6	−166.4 (4)	C9—C10—C11—C12	98.2 (5)
C3—C4—C5—O1	177.5 (4)	C16—C11—C12—C13	−0.1 (6)
N1—C4—C5—O1	−1.7 (5)	C10—C11—C12—C13	−179.5 (4)
C3—C4—C5—C6	−0.2 (6)	C11—C12—C13—C14	1.1 (7)
N1—C4—C5—C6	−179.5 (4)	C12—C13—C14—C15	−1.6 (8)
C2—C1—C6—C5	−0.8 (7)	C13—C14—C15—C16	1.1 (8)
Cl1—C1—C6—C5	−179.2 (3)	C12—C11—C16—C15	−0.4 (7)
O1—C5—C6—C1	−177.4 (4)	C10—C11—C16—C15	179.0 (4)
C4—C5—C6—C1	0.5 (6)	C14—C15—C16—C11	−0.1 (7)
C5—O1—C7—C8	−18.3 (5)		

## References

[bb1] Bruker (2005). *SMART*, *SAINT *and *SADABS* Bruker AXS Inc., Madison, Wisconsin, USA.

[bb2] Flack, H. D. (1983). *Acta Cryst.* A**39**, 876–881.

[bb3] Li, Z.-B., Luo, Y.-H., Dong, W.-L., Li, J. & Zuo, H. (2008). *Acta Cryst.* E**64**, o1610. 10.1107/S1600536808022514 PMC2962222 21203303

[bb4] Sheldrick, G. M. (2008). *Acta Cryst.* A**64**, 112–122. 10.1107/S0108767307043930 18156677

[bb5] Zuo, H., Meng, L., Ghate, M., Hwang, K. H., Cho, Y. K., Chandrasekhar, S., Reddy, C. R. & Shin, D. S. (2008). *Tetrahedron Lett.* **49**, 3827–3830.

